# Supplementary material for: Nest defence behavioural reaction norms: testing life-history and parental investment theory predictions
Source: R Soc Open Sci. 2019 Apr 10;6(4):182180. doi: 10.1098/rsos.182180 (PMC6502369; doi:10.1098/rsos.182180)
Supplement: Prior specification mixed models and supplementary tables [file rsos182180supp1.docx]

**SUPPLEMENTARY MATERIAL**

**Nest defence behavioural reaction norms: testing life history and parental investment theory predictions**

Bert Thys, Yorick Lambreghts, Rianne Pinxten, Marcel Eens

Contents:

- Prior specifications mixed models
- Table S1: GLM predicting nest success
- Table S2: Covariance matrix among (ID) and within (R) individuals
- Table S3: Dataset supporting the manuscript

**Prior specifications mixed models**

Mixed models were run using the MCMCglmm package in R [1]. Models ran for 2 200 000 iterations with a burn-in of 200 000 and a thinning-interval of 1000 iterations, and the following prior specifications:

(1) Prior specification for random regression model:

prior.RR = list(R=list(V=1, nu=1), G=list(G1=list(V=diag(2), nu=2, alpha.mu=c(0,0), alpha.V=diag(2)*1000)))

(2) Prior specification for multivariate mixed model:

prior.Multi = list(R=list(V=diag(c(1,0.0001,0.0001,0.0001,0.0001)), nu=0.005, fix=2),

G=list(G1=list(V=diag(5), nu=0.005)))

**Table S1**: Results of GLM with logit-link predicting nest success in female great tits (N=104). Posterior mean estimates (β) are given with 95% credible intervals. Nest defence represents best linear unbiased predictors of the random intercepts for individuals, extracted from the random regression model presented in Table 1 of the main text.

|  | **β (CrI)** |
| --- | --- |
| Intercept | 1.34 (0.86 ; 1.83) |
| Lay date | -0.08 (-0.57 ; 0.44) |
| Clutch size | -0.20 (-0.74 ; 0.33) |
| Nest defence | -0.23 (-0.78 ; 0.33) |

**Table S2:** Posterior mean estimates of among-individual (ID) and within-individual (R) variances and covariances between hissing behaviour (Nest Defence), Julian lay date (JLD), clutch size, fledgling number (Fl number) and fledgling mass (Fl mass). Variances are given on the diagonal and covariances (with CrI) between traits below the diagonal. Given the absence of repeated measurements for lay date, clutch size, fledgling number and fledgling mass, within-individual variances involving these traits were set to be essentially zero (see prior specification multivariate mixed model), and hence within-individual covariances were not estimated [2,3].

| **ID** | Nest defence | JLD | Clutch size | Fl number | Fl mass |
| --- | --- | --- | --- | --- | --- |
| Nest defence | 0.78 |  |  |  |  |
| JLD | -0.15  (-0.39 ; 0.08) | 1.03 |  |  |  |
| Clutch size | -0.09  (-0.31 ; 0.15) | -0.66  (-0.98 ; -0.39) | 1.01 |  |  |
| Fl number | -0.02  (-0.24 ; 0.23) | -0.52  (-0.82 ; -0.25) | 0.78  (0.49 ; 1.10) | 1.03 |  |
| Fl mass | 0.12  (-0.11 ; 0.35) | 0.12  (-0.16 ; 0.38) | -0.24  (-0.48 ; 0.02) | -0.08  (-0.36 ; 0.18) | 1.00 |
|  |  |  |  |  |  |
| **R** | Nest defence | JLD | Clutch size | Fl number | Fl mass |
| Nest defence | 0.26 |  |  |  |  |
| JLD |  | 0.0001 |  |  |  |
| Clutch size |  |  | 0.0001 |  |  |
| Fl number |  |  |  | 0.0001 |  |
| Fl mass |  |  |  |  | 0.0001 |

**References**

1. Hadfield JD. 2010 MCMC methods for multi-response generalized linear mixed models: The MCMCglmm R package. *J Stat Softw*. 33, 1-22. (doi:10.1002/ana.22635)
2. Dingemanse NJ, Dochtermann NA. 2013 Quantifying individual variation in behaviour: Mixed-effect modelling approaches. *J Anim Ecol*. 82, 39-54. (doi:10.1111/1365-2656.12013)
3. Houslay TM, Wilson AJ. 2017 Avoiding the misuse of BLUP in behavioural ecology. *Behav Ecol*. 28, 948-952. (doi:10.1093/beheco/arx023)

**Table S3**: Dataset supporting the article. **ID** = unique female identity; **Age** = female age; **HissCalls** = number of hissing calls produced; **NestCycle** = day in the nesting cycle of the test, centered around hatching day (=0); **meanTime** = mean time of day of testing per individual, in minutes after sunrise; **devTime** = time of day of the test, in minutes after sunrise and expressed as the deviation of each observation from an individuals’ mean test time; **JLD** = Julian lay date; **Clutch** = clutch size; **NS** = nest success, yes (y) or no (n); **Fl number** = number of fledglings; **Fl mass** = average fledgling mass.

| ID | Age | HissCalls | NestCycle | meanTime | devTime | JLD | Clutch | NS | Fl number | Fl Mass |
| --- | --- | --- | --- | --- | --- | --- | --- | --- | --- | --- |
| 1 | 1 | 15 | -13 | 335.75 | -66,75 | -80 | 6 | y | 6 | 17,12 |
| 1 | 1 | 19 | -10 | 335.75 | 171,25 | -80 | 6 | y | 6 | 17,12 |
| 1 | 1 | 17 | -6 | 335.75 | 56,25 | -80 | 6 | y | 6 | 17,12 |
| 1 | 1 | 20 | 1 | 335.75 | -160,75 | -80 | 6 | y | 6 | 17,12 |
| 4 | 1 | 0 | -10 | 344.6 | -85,6 | -72 | 7 | y | 6 | 16,88 |
| 4 | 1 | 5 | -7 | 344.6 | -77,6 | -72 | 7 | y | 6 | 16,88 |
| 4 | 1 | 8 | -3 | 344.6 | 59,4 | -72 | 7 | y | 6 | 16,88 |
| 4 | 1 | 7 | 2 | 344.6 | -174,6 | -72 | 7 | y | 6 | 16,88 |
| 4 | 1 | 12 | 5 | 344.6 | 278,4 | -72 | 7 | y | 6 | 16,88 |
| 5 | 3 | 0 | -11 | 230 | 0 | -68 | 7 | n | 0 | NA |
| 6 | 1 | 17 | -10 | 427.6 | 87,4 | -78 | 4 | y | 4 | 18,28 |
| 6 | 1 | 7 | -7 | 427.6 | 172,4 | -78 | 4 | y | 4 | 18,28 |
| 6 | 1 | 19 | -3 | 427.6 | -52,6 | -78 | 4 | y | 4 | 18,28 |
| 6 | 1 | 22 | 1 | 427.6 | -139,6 | -78 | 4 | y | 4 | 18,28 |
| 6 | 1 | 21 | 4 | 427.6 | -67,6 | -78 | 4 | y | 4 | 18,28 |
| 7 | 2 | 0 | -10 | 366 | -143 | -82 | 10 | y | 9 | 16,27 |
| 7 | 2 | 0 | -7 | 366 | 76 | -82 | 10 | y | 9 | 16,27 |
| 7 | 2 | 8 | -3 | 366 | -54 | -82 | 10 | y | 9 | 16,27 |
| 7 | 2 | 3 | 1 | 366 | -36 | -82 | 10 | y | 9 | 16,27 |
| 7 | 2 | 8 | 4 | 366 | 157 | -82 | 10 | y | 9 | 16,27 |
| 10 | 1 | 2 | -10 | 275.25 | 73,75 | -72 | 6 | y | 6 | 16,85 |
| 10 | 1 | 3 | -7 | 275.25 | -5,25 | -72 | 6 | y | 6 | 16,85 |
| 10 | 1 | 7 | -3 | 275.25 | 53,75 | -72 | 6 | y | 6 | 16,85 |
| 10 | 1 | 2 | 1 | 275.25 | -122,25 | -72 | 6 | y | 6 | 16,85 |
| 11 | 2 | 13 | -12 | 372.5 | 125,5 | -79 | 7 | y | 7 | 16,14 |
| 11 | 2 | 13 | -9 | 372.5 | -82,5 | -79 | 7 | y | 7 | 16,14 |
| 11 | 2 | 17 | -5 | 372.5 | 88,5 | -79 | 7 | y | 7 | 16,14 |
| 11 | 2 | 18 | 1 | 372.5 | -131,5 | -79 | 7 | y | 7 | 16,14 |
| 13 | 1 | 7 | -13 | 469.5 | 101,5 | -82 | 9 | y | 9 | 16,74 |
| 13 | 1 | 10 | -10 | 469.5 | -25,5 | -82 | 9 | y | 9 | 16,74 |
| 13 | 1 | 15 | -6 | 469.5 | -47,5 | -82 | 9 | y | 9 | 16,74 |
| 13 | 1 | 13 | 1 | 469.5 | -28,5 | -82 | 9 | y | 9 | 16,74 |
| 14 | 1 | 2 | -11 | 399.25 | 129,75 | -67 | 6 | y | 2 | 14,00 |
| 14 | 1 | 0 | -8 | 399.25 | -175,25 | -67 | 6 | y | 2 | 14,00 |
| 14 | 1 | 5 | -5 | 399.25 | -152,25 | -67 | 6 | y | 2 | 14,00 |
| 14 | 1 | 0 | 1 | 399.25 | 197,75 | -67 | 6 | y | 2 | 14,00 |
| 16 | 2 | 0 | -13 | 364 | 20 | -80 | 7 | y | 4 | 18,15 |
| 16 | 2 | 1 | -10 | 364 | 26 | -80 | 7 | y | 4 | 18,15 |
| 16 | 2 | 0 | -6 | 364 | 104 | -80 | 7 | y | 4 | 18,15 |
| 16 | 2 | 3 | 1 | 364 | -150 | -80 | 7 | y | 4 | 18,15 |
| 17 | 2 | 15 | -13 | 322 | -26 | -83 | 7 | y | 7 | 15,17 |
| 17 | 2 | 10 | -9 | 322 | 219 | -83 | 7 | y | 7 | 15,17 |
| 17 | 2 | 30 | -6 | 322 | -65 | -83 | 7 | y | 7 | 15,17 |
| 17 | 2 | 33 | 1 | 322 | -58 | -83 | 7 | y | 7 | 15,17 |
| 17 | 2 | 35 | 4 | 322 | -70 | -83 | 7 | y | 7 | 15,17 |
| 20 | 1 | 0 | -12 | 208.5 | -39,5 | -80 | 6 | y | 5 | 17,42 |
| 20 | 1 | 5 | -9 | 208.5 | 54,5 | -80 | 6 | y | 5 | 17,42 |
| 20 | 1 | 16 | -5 | 208.5 | 40,5 | -80 | 6 | y | 5 | 17,42 |
| 20 | 1 | 5 | 1 | 208.5 | -55,5 | -80 | 6 | y | 5 | 17,42 |
| 23 | 1 | 0 | -13 | 319.25 | -69,25 | -78 | 7 | y | 7 | 16,46 |
| 23 | 1 | 4 | -10 | 319.25 | 41,75 | -78 | 7 | y | 7 | 16,46 |
| 23 | 1 | 0 | -6 | 319.25 | 112,75 | -78 | 7 | y | 7 | 16,46 |
| 23 | 1 | 1 | 1 | 319.25 | -85,25 | -78 | 7 | y | 7 | 16,46 |
| 25 | 1 | 1 | -11 | 272 | 0 | -86 | 6 | n | 0 | NA |
| 29 | 1 | 15 | -7 | 396.75 | 251,25 | -83 | 10 | n | 0 | NA |
| 29 | 1 | 20 | -4 | 396.75 | 16,25 | -83 | 10 | n | 0 | NA |
| 29 | 1 | 16 | 1 | 396.75 | -141,75 | -83 | 10 | n | 0 | NA |
| 29 | 1 | 9 | 4 | 396.75 | -125,75 | -83 | 10 | n | 0 | NA |
| 30 | 2 | 2 | -12 | 420.4 | 20,6 | -87 | 11 | y | 11 | 15,97 |
| 30 | 2 | 1 | -9 | 420.4 | 132,6 | -87 | 11 | y | 11 | 15,97 |
| 30 | 2 | 0 | -5 | 420.4 | -0,4 | -87 | 11 | y | 11 | 15,97 |
| 30 | 2 | 3 | 1 | 420.4 | -9,4 | -87 | 11 | y | 11 | 15,97 |
| 30 | 2 | 12 | 4 | 420.4 | -143,4 | -87 | 11 | y | 11 | 15,97 |
| 31 | 2 | 0 | -11 | 285.5 | -43,5 | -81 | 8 | y | 8 | 15,68 |
| 31 | 2 | 2 | -8 | 285.5 | -26,5 | -81 | 8 | y | 8 | 15,68 |
| 31 | 2 | 1 | -4 | 285.5 | 132,5 | -81 | 8 | y | 8 | 15,68 |
| 31 | 2 | 3 | 1 | 285.5 | -62,5 | -81 | 8 | y | 8 | 15,68 |
| 32 | 1 | 5 | -12 | 342.5 | -37,5 | -85 | 9 | y | 8 | 15,49 |
| 32 | 1 | 14 | -9 | 342.5 | 42,5 | -85 | 9 | y | 8 | 15,49 |
| 32 | 1 | 4 | -5 | 342.5 | 48,5 | -85 | 9 | y | 8 | 15,49 |
| 32 | 1 | 8 | 1 | 342.5 | -53,5 | -85 | 9 | y | 8 | 15,49 |
| 33 | 2 | 7 | -13 | 308.4 | 77,6 | -78 | 7 | y | 6 | 16,28 |
| 33 | 2 | 6 | -10 | 308.4 | 82,6 | -78 | 7 | y | 6 | 16,28 |
| 33 | 2 | 0 | -5 | 308.4 | -22,4 | -78 | 7 | y | 6 | 16,28 |
| 33 | 2 | 7 | 1 | 308.4 | -44,4 | -78 | 7 | y | 6 | 16,28 |
| 33 | 2 | 17 | 4 | 308.4 | -93,4 | -78 | 7 | y | 6 | 16,28 |
| 36 | 1 | 0 | -12 | 431.5 | -38,5 | -82 | 8 | y | 8 | 18,53 |
| 36 | 1 | 1 | -9 | 431.5 | 39,5 | -82 | 8 | y | 8 | 18,53 |
| 36 | 1 | 6 | -5 | 431.5 | -34,5 | -82 | 8 | y | 8 | 18,53 |
| 36 | 1 | 10 | 1 | 431.5 | 33,5 | -82 | 8 | y | 8 | 18,53 |
| 38 | 1 | 16 | -10 | 413.2 | -15,2 | -79 | 5 | y | 4 | 17,45 |
| 38 | 1 | 24 | -7 | 413.2 | -13,2 | -79 | 5 | y | 4 | 17,45 |
| 38 | 1 | 29 | -3 | 413.2 | 183,8 | -79 | 5 | y | 4 | 17,45 |
| 38 | 1 | 37 | 1 | 413.2 | 105,8 | -79 | 5 | y | 4 | 17,45 |
| 38 | 1 | 38 | 4 | 413.2 | -261,2 | -79 | 5 | y | 4 | 17,45 |
| 40 | 2 | 4 | -11 | 303 | 175 | -82 | 6 | y | 5 | 18,28 |
| 40 | 2 | 5 | -8 | 303 | -61 | -82 | 6 | y | 5 | 18,28 |
| 40 | 2 | 4 | -4 | 303 | -23 | -82 | 6 | y | 5 | 18,28 |
| 40 | 2 | 5 | 1 | 303 | -91 | -82 | 6 | y | 5 | 18,28 |
| 43 | 1 | 16 | -10 | 206 | -10 | -77 | 9 | n | 0 | NA |
| 43 | 1 | 17 | -7 | 206 | -88 | -77 | 9 | n | 0 | NA |
| 43 | 1 | 5 | -3 | 206 | -54 | -77 | 9 | n | 0 | NA |
| 43 | 1 | 12 | 1 | 206 | 13 | -77 | 9 | n | 0 | NA |
| 43 | 1 | 8 | 4 | 206 | 139 | -77 | 9 | n | 0 | NA |
| 44 | 1 | 20 | -9 | 431 | 69 | -81 | 7 | y | 5 | 17,52 |
| 44 | 1 | 19 | -6 | 431 | 159 | -81 | 7 | y | 5 | 17,52 |
| 44 | 1 | 22 | -2 | 431 | 38 | -81 | 7 | y | 5 | 17,52 |
| 44 | 1 | 23 | 1 | 431 | -266 | -81 | 7 | y | 5 | 17,52 |
| 45 | 1 | 26 | -12 | 317.5 | 6,5 | -82 | 8 | y | 8 | 16,48 |
| 45 | 1 | 7 | -9 | 317.5 | -53,5 | -82 | 8 | y | 8 | 16,48 |
| 45 | 1 | 24 | -5 | 317.5 | 23,5 | -82 | 8 | y | 8 | 16,48 |
| 45 | 1 | 18 | 1 | 317.5 | 23,5 | -82 | 8 | y | 8 | 16,48 |
| 46 | 2 | 28 | -12 | 322 | 7 | -82 | 7 | y | 4 | 17,05 |
| 46 | 2 | 12 | -9 | 322 | -64 | -82 | 7 | y | 4 | 17,05 |
| 46 | 2 | 28 | -5 | 322 | 30 | -82 | 7 | y | 4 | 17,05 |
| 46 | 2 | 35 | 1 | 322 | 27 | -82 | 7 | y | 4 | 17,05 |
| 47 | 1 | 22 | -12 | 323.75 | 30,25 | -88 | 10 | y | 9 | 16,54 |
| 47 | 1 | 21 | -9 | 323.75 | -99,75 | -88 | 10 | y | 9 | 16,54 |
| 47 | 1 | 35 | -5 | 323.75 | -53,75 | -88 | 10 | y | 9 | 16,54 |
| 47 | 1 | 24 | 2 | 323.75 | 123,25 | -88 | 10 | y | 9 | 16,54 |
| 48 | 1 | 35 | -9 | 494 | -50 | -80 | 9 | n | 0 | NA |
| 48 | 1 | 39 | -6 | 494 | 59 | -80 | 9 | n | 0 | NA |
| 48 | 1 | 40 | -2 | 494 | -9 | -80 | 9 | n | 0 | NA |
| 49 | 1 | 2 | -13 | 325.75 | 25,25 | -85 | 9 | y | 9 | 17,43 |
| 49 | 1 | 4 | -10 | 325.75 | -41,75 | -85 | 9 | y | 9 | 17,43 |
| 49 | 1 | 7 | -6 | 325.75 | -6,75 | -85 | 9 | y | 9 | 17,43 |
| 49 | 1 | 10 | 1 | 325.75 | 23,25 | -85 | 9 | y | 9 | 17,43 |
| 50 | 1 | 24 | -12 | 348.8 | 83,2 | -80 | 5 | y | 5 | 19,82 |
| 50 | 1 | 36 | -9 | 348.8 | -90,8 | -80 | 5 | y | 5 | 19,82 |
| 50 | 1 | 36 | -5 | 348.8 | -123,8 | -80 | 5 | y | 5 | 19,82 |
| 50 | 1 | 36 | 1 | 348.8 | 218,2 | -80 | 5 | y | 5 | 19,82 |
| 50 | 1 | 37 | 4 | 348.8 | -86,8 | -80 | 5 | y | 5 | 19,82 |
| 51 | 1 | 0 | -10 | 407.25 | 118,75 | -87 | 8 | y | 7 | 17,27 |
| 51 | 1 | 0 | -7 | 407.25 | -126,25 | -87 | 8 | y | 7 | 17,27 |
| 51 | 1 | 0 | -3 | 407.25 | 67,75 | -87 | 8 | y | 7 | 17,27 |
| 51 | 1 | 2 | 1 | 407.25 | -60,25 | -87 | 8 | y | 7 | 17,27 |
| 52 | 2 | 11 | -10 | 351.6 | 69,4 | -86 | 9 | y | 9 | 16,09 |
| 52 | 2 | 7 | -7 | 351.6 | -117,6 | -86 | 9 | y | 9 | 16,09 |
| 52 | 2 | 6 | -3 | 351.6 | -25,6 | -86 | 9 | y | 9 | 16,09 |
| 52 | 2 | 16 | 1 | 351.6 | -35,6 | -86 | 9 | y | 9 | 16,09 |
| 52 | 2 | 23 | 4 | 351.6 | 109,4 | -86 | 9 | y | 9 | 16,09 |
| 66 | 2 | 2 | -12 | 285.5 | 78,5 | -69 | 8 | n | 0 | NA |
| 66 | 2 | 1 | -9 | 285.5 | -3,5 | -69 | 8 | n | 0 | NA |
| 66 | 2 | 0 | -5 | 285.5 | 46,5 | -69 | 8 | n | 0 | NA |
| 66 | 2 | 6 | 1 | 285.5 | -121,5 | -69 | 8 | n | 0 | NA |
| 67 | 1 | 0 | -12 | 391.5 | -27,5 | -87 | 7 | y | 7 | 16,73 |
| 67 | 1 | 9 | -9 | 391.5 | -44,5 | -87 | 7 | y | 7 | 16,73 |
| 67 | 1 | 1 | -5 | 391.5 | -71,5 | -87 | 7 | y | 7 | 16,73 |
| 67 | 1 | 6 | 2 | 391.5 | 143,5 | -87 | 7 | y | 7 | 16,73 |
| 68 | 2 | 0 | -11 | 349 | -4 | -87 | 7 | y | 7 | 16,86 |
| 68 | 2 | 17 | -8 | 349 | -112 | -87 | 7 | y | 7 | 16,86 |
| 68 | 2 | 20 | -4 | 349 | -57 | -87 | 7 | y | 7 | 16,86 |
| 68 | 2 | 18 | 1 | 349 | 112 | -87 | 7 | y | 7 | 16,86 |
| 68 | 2 | 21 | 4 | 349 | 61 | -87 | 7 | y | 7 | 16,86 |
| 85 | 2 | 0 | -11 | 268.4 | -138,4 | -80 | 4 | y | 4 | 16,75 |
| 85 | 2 | 0 | -8 | 268.4 | 50,6 | -80 | 4 | y | 4 | 16,75 |
| 85 | 2 | 1 | -4 | 268.4 | 10,6 | -80 | 4 | y | 4 | 16,75 |
| 85 | 2 | 4 | 1 | 268.4 | -129,4 | -80 | 4 | y | 4 | 16,75 |
| 85 | 2 | 1 | 4 | 268.4 | 206,6 | -80 | 4 | y | 4 | 16,75 |
| 86 | 1 | 8 | -9 | 345 | 341 | -76 | 4 | y | 4 | 15,30 |
| 86 | 1 | 0 | -6 | 345 | 104 | -76 | 4 | y | 4 | 15,30 |
| 86 | 1 | 9 | -3 | 345 | -145 | -76 | 4 | y | 4 | 15,30 |
| 86 | 1 | 11 | 1 | 345 | -168 | -76 | 4 | y | 4 | 15,30 |
| 86 | 1 | 10 | 4 | 345 | -132 | -76 | 4 | y | 4 | 15,30 |
| 87 | 1 | 6 | -11 | 287.4 | 2,6 | -79 | 7 | y | 4 | 18,65 |
| 87 | 1 | 3 | -8 | 287.4 | 175,6 | -79 | 7 | y | 4 | 18,65 |
| 87 | 1 | 3 | -4 | 287.4 | -73,4 | -79 | 7 | y | 4 | 18,65 |
| 87 | 1 | 0 | 2 | 287.4 | -69,4 | -79 | 7 | y | 4 | 18,65 |
| 87 | 1 | 6 | 4 | 287.4 | -35,4 | -79 | 7 | y | 4 | 18,65 |
| 89 | 1 | 11 | -10 | 378.2 | -31,2 | -82 | 7 | y | 7 | 17,03 |
| 89 | 1 | 3 | -7 | 378.2 | -30,2 | -82 | 7 | y | 7 | 17,03 |
| 89 | 1 | 16 | -3 | 378.2 | 159,8 | -82 | 7 | y | 7 | 17,03 |
| 89 | 1 | 24 | 1 | 378.2 | -90,2 | -82 | 7 | y | 7 | 17,03 |
| 89 | 1 | 4 | 4 | 378.2 | -8,2 | -82 | 7 | y | 7 | 17,03 |
| 93 | 1 | 7 | -10 | 420.4 | -95,4 | -83 | 8 | y | 8 | 13,99 |
| 93 | 1 | 2 | -7 | 420.4 | -101,4 | -83 | 8 | y | 8 | 13,99 |
| 93 | 1 | 10 | -3 | 420.4 | 124,6 | -83 | 8 | y | 8 | 13,99 |
| 93 | 1 | 8 | 1 | 420.4 | 10,6 | -83 | 8 | y | 8 | 13,99 |
| 93 | 1 | 0 | 4 | 420.4 | 61,6 | -83 | 8 | y | 8 | 13,99 |
| 94 | 2 | 13 | -10 | 263.5 | 36,5 | -76 | 8 | y | 7 | 15,36 |
| 94 | 2 | 6 | -7 | 263.5 | 127,5 | -76 | 8 | y | 7 | 15,36 |
| 94 | 2 | 16 | -4 | 263.5 | -42,5 | -76 | 8 | y | 7 | 15,36 |
| 94 | 2 | 14 | 1 | 263.5 | -121,5 | -76 | 8 | y | 7 | 15,36 |
| 96 | 1 | 12 | -11 | 396 | 143 | -73 | 5 | y | 4 | 16,30 |
| 96 | 1 | 19 | -8 | 396 | 42 | -73 | 5 | y | 4 | 16,30 |
| 96 | 1 | 10 | -4 | 396 | -198 | -73 | 5 | y | 4 | 16,30 |
| 96 | 1 | 6 | 1 | 396 | 13 | -73 | 5 | y | 4 | 16,30 |
| 97 | 2 | 6 | -13 | 296.8 | 12,2 | -76 | 8 | y | 9 | 16,08 |
| 97 | 2 | 11 | -10 | 296.8 | 246,2 | -76 | 8 | y | 9 | 16,08 |
| 97 | 2 | 16 | -6 | 296.8 | -26,8 | -76 | 8 | y | 9 | 16,08 |
| 97 | 2 | 15 | 1 | 296.8 | -69,8 | -76 | 8 | y | 9 | 16,08 |
| 97 | 2 | 16 | 4 | 296.8 | -161,8 | -76 | 8 | y | 9 | 16,08 |
| 99 | 1 | 14 | -10 | 501.8 | -54,8 | -86 | 10 | y | 8 | 16,40 |
| 99 | 1 | 12 | -7 | 501.8 | -212,8 | -86 | 10 | y | 8 | 16,40 |
| 99 | 1 | 1 | -3 | 501.8 | -82,8 | -86 | 10 | y | 8 | 16,40 |
| 99 | 1 | 2 | 2 | 501.8 | 131,2 | -86 | 10 | y | 8 | 16,40 |
| 99 | 1 | 0 | 4 | 501.8 | 219,2 | -86 | 10 | y | 8 | 16,40 |
| 100 | 1 | 3 | -7 | 567.67 | -91,67 | -85 | 8 | y | 2 | 13,40 |
| 100 | 1 | 4 | -4 | 567.67 | 49,33 | -85 | 8 | y | 2 | 13,40 |
| 100 | 1 | 4 | 1 | 567.67 | 69,33 | -85 | 8 | y | 2 | 13,40 |
| 101 | 2 | 26 | -11 | 469.8 | -74,8 | -86 | 7 | y | 6 | 17,43 |
| 101 | 2 | 25 | -8 | 469.8 | -203,8 | -86 | 7 | y | 6 | 17,43 |
| 101 | 2 | 32 | -4 | 469.8 | 146,2 | -86 | 7 | y | 6 | 17,43 |
| 101 | 2 | 31 | 1 | 469.8 | -21,8 | -86 | 7 | y | 6 | 17,43 |
| 101 | 2 | 34 | 4 | 469.8 | 154,2 | -86 | 7 | y | 6 | 17,43 |
| 102 | 1 | 1 | -12 | 338.5 | -10,5 | -87 | 6 | n | 0 | NA |
| 102 | 1 | 0 | -9 | 338.5 | -66,5 | -87 | 6 | n | 0 | NA |
| 102 | 1 | 5 | -5 | 338.5 | 105,5 | -87 | 6 | n | 0 | NA |
| 102 | 1 | 0 | 1 | 338.5 | -28,5 | -87 | 6 | n | 0 | NA |
| 104 | 1 | 20 | -17 | 390.4 | -90,4 | -89 | 7 | y | 7 | 15,61 |
| 104 | 1 | 19 | -14 | 390.4 | -112,4 | -89 | 7 | y | 7 | 15,61 |
| 104 | 1 | 25 | -10 | 390.4 | 148,6 | -89 | 7 | y | 7 | 15,61 |
| 104 | 1 | 15 | 1 | 390.4 | -113,4 | -89 | 7 | y | 7 | 15,61 |
| 104 | 1 | 26 | 4 | 390.4 | 167,6 | -89 | 7 | y | 7 | 15,61 |
| 106 | 2 | 0 | -13 | 305 | -95 | -85 | 8 | y | 7 | 17,76 |
| 106 | 2 | 0 | -10 | 305 | -66 | -85 | 8 | y | 7 | 17,76 |
| 106 | 2 | 0 | -6 | 305 | -65 | -85 | 8 | y | 7 | 17,76 |
| 106 | 2 | 0 | 1 | 305 | -32 | -85 | 8 | y | 7 | 17,76 |
| 106 | 2 | 5 | 4 | 305 | 258 | -85 | 8 | y | 7 | 17,76 |
| 107 | 2 | 0 | -11 | 303.8 | -31,8 | -88 | 10 | n | 0 | NA |
| 107 | 2 | 7 | -8 | 303.8 | 57,2 | -88 | 10 | n | 0 | NA |
| 107 | 2 | 1 | -4 | 303.8 | -117,8 | -88 | 10 | n | 0 | NA |
| 107 | 2 | 1 | 1 | 303.8 | 133,2 | -88 | 10 | n | 0 | NA |
| 107 | 2 | 0 | 4 | 303.8 | -40,8 | -88 | 10 | n | 0 | NA |
| 108 | 1 | 4 | -14 | 263.6 | 17,4 | -84 | 7 | y | 2 | 15,90 |
| 108 | 1 | 19 | -11 | 263.6 | -29,6 | -84 | 7 | y | 2 | 15,90 |
| 108 | 1 | 25 | -7 | 263.6 | -18,6 | -84 | 7 | y | 2 | 15,90 |
| 108 | 1 | 15 | 1 | 263.6 | 38,4 | -84 | 7 | y | 2 | 15,90 |
| 108 | 1 | 12 | 4 | 263.6 | -7,6 | -84 | 7 | y | 2 | 15,90 |
| 110 | 2 | 21 | -10 | 369.2 | 5,8 | -93 | 7 | y | 6 | NA |
| 110 | 2 | 23 | -7 | 369.2 | -72,2 | -93 | 7 | y | 6 | NA |
| 110 | 2 | 15 | -3 | 369.2 | -10,2 | -93 | 7 | y | 6 | NA |
| 110 | 2 | 7 | 1 | 369.2 | -142,2 | -93 | 7 | y | 6 | NA |
| 110 | 2 | 17 | 4 | 369.2 | 218,8 | -93 | 7 | y | 6 | NA |
| 111 | 1 | 5 | -17 | 438 | -57 | -91 | 9 | n | 0 | NA |
| 111 | 1 | 3 | -10 | 438 | -74 | -91 | 9 | n | 0 | NA |
| 111 | 1 | 1 | 1 | 438 | 131 | -91 | 9 | n | 0 | NA |
| 112 | 2 | 28 | -13 | 312.75 | 62,25 | -82 | 9 | y | 8 | 14,93 |
| 112 | 2 | 17 | -10 | 312.75 | -76,75 | -82 | 9 | y | 8 | 14,93 |
| 112 | 2 | 24 | -6 | 312.75 | 130,25 | -82 | 9 | y | 8 | 14,93 |
| 112 | 2 | 29 | 2 | 312.75 | -115,75 | -82 | 9 | y | 8 | 14,93 |
| 113 | 1 | 27 | -7 | 205.67 | 148,33 | -74 | 4 | y | 3 | 15,77 |
| 113 | 1 | 28 | -4 | 205.67 | -22,67 | -74 | 4 | y | 3 | 15,77 |
| 113 | 1 | 26 | 1 | 205.67 | -125,67 | -74 | 4 | y | 3 | 15,77 |
| 117 | 1 | 0 | -10 | 295 | -33 | -80 | 6 | y | 5 | 15,80 |
| 117 | 1 | 0 | -7 | 295 | -24 | -80 | 6 | y | 5 | 15,80 |
| 117 | 1 | 0 | -3 | 295 | -28 | -80 | 6 | y | 5 | 15,80 |
| 117 | 1 | 0 | 1 | 295 | 85 | -80 | 6 | y | 5 | 15,80 |
| 118 | 1 | 30 | -10 | 204 | -60 | -79 | 6 | y | 5 | 16,28 |
| 118 | 1 | 34 | -7 | 204 | 107 | -79 | 6 | y | 5 | 16,28 |
| 118 | 1 | 38 | -2 | 204 | 21 | -79 | 6 | y | 5 | 16,28 |
| 118 | 1 | 22 | 1 | 204 | -68 | -79 | 6 | y | 5 | 16,28 |
| 119 | 3 | 7 | -10 | 228.2 | 213,8 | -76 | 7 | n | 0 | NA |
| 119 | 3 | 14 | -7 | 228.2 | -64,2 | -76 | 7 | n | 0 | NA |
| 119 | 3 | 13 | -3 | 228.2 | 52,8 | -76 | 7 | n | 0 | NA |
| 119 | 3 | 19 | 1 | 228.2 | -39,2 | -76 | 7 | n | 0 | NA |
| 119 | 3 | 12 | 4 | 228.2 | -163,2 | -76 | 7 | n | 0 | NA |
| 120 | 1 | 0 | -11 | 239.25 | -18,25 | -78 | 6 | y | 6 | 15,62 |
| 120 | 1 | 12 | -8 | 239.25 | 18,75 | -78 | 6 | y | 6 | 15,62 |
| 120 | 1 | 19 | -4 | 239.25 | 108,75 | -78 | 6 | y | 6 | 15,62 |
| 120 | 1 | 12 | 1 | 239.25 | -109,25 | -78 | 6 | y | 6 | 15,62 |
| 121 | 3 | 20 | -11 | 236 | 70 | -82 | 8 | n | 0 | NA |
| 121 | 3 | 13 | -8 | 236 | -105 | -82 | 8 | n | 0 | NA |
| 121 | 3 | 19 | -4 | 236 | 35 | -82 | 8 | n | 0 | NA |
| 124 | 1 | 27 | -11 | 308.8 | 109,2 | -86 | 8 | y | 7 | 16,80 |
| 124 | 1 | 27 | -8 | 308.8 | 0,2 | -86 | 8 | y | 7 | 16,80 |
| 124 | 1 | 25 | -4 | 308.8 | -102,8 | -86 | 8 | y | 7 | 16,80 |
| 124 | 1 | 18 | 1 | 308.8 | 113,2 | -86 | 8 | y | 7 | 16,80 |
| 124 | 1 | 26 | 4 | 308.8 | -119,8 | -86 | 8 | y | 7 | 16,80 |
| 126 | 3 | 32 | -12 | 391.6 | -51,6 | -82 | 9 | n | 0 | NA |
| 126 | 3 | 29 | -9 | 391.6 | -179,6 | -82 | 9 | n | 0 | NA |
| 126 | 3 | 33 | -4 | 391.6 | 42,4 | -82 | 9 | n | 0 | NA |
| 126 | 3 | 27 | 1 | 391.6 | 295,4 | -82 | 9 | n | 0 | NA |
| 126 | 3 | 19 | 4 | 391.6 | -106,6 | -82 | 9 | n | 0 | NA |
| 128 | 1 | 21 | -11 | 266.25 | -82,25 | -83 | 8 | y | 3 | 16,80 |
| 128 | 1 | 18 | -8 | 266.25 | 35,75 | -83 | 8 | y | 3 | 16,80 |
| 128 | 1 | 22 | -4 | 266.25 | -65,25 | -83 | 8 | y | 3 | 16,80 |
| 128 | 1 | 3 | 1 | 266.25 | 111,75 | -83 | 8 | y | 3 | 16,80 |
| 129 | 1 | 0 | -12 | 275.5 | 66,5 | -79 | 7 | n | 0 | NA |
| 129 | 1 | 5 | -9 | 275.5 | 49,5 | -79 | 7 | n | 0 | NA |
| 129 | 1 | 9 | -5 | 275.5 | 3,5 | -79 | 7 | n | 0 | NA |
| 129 | 1 | 16 | 1 | 275.5 | -119,5 | -79 | 7 | n | 0 | NA |
| 130 | 1 | 18 | -13 | 225.33 | -45,33 | -80 | 6 | n | 0 | NA |
| 130 | 1 | 14 | -10 | 225.33 | 73,67 | -80 | 6 | n | 0 | NA |
| 130 | 1 | 16 | -6 | 225.33 | -28,33 | -80 | 6 | n | 0 | NA |
| 132 | 1 | 0 | -12 | 293 | 20 | -86 | 7 | y | 7 | 14,50 |
| 132 | 1 | 25 | -9 | 293 | -7 | -86 | 7 | y | 7 | 14,50 |
| 132 | 1 | 10 | -5 | 293 | -143 | -86 | 7 | y | 7 | 14,50 |
| 132 | 1 | 18 | 1 | 293 | 130 | -86 | 7 | y | 7 | 14,50 |
| 133 | 2 | 38 | -12 | 370.4 | 60,6 | -92 | 9 | n | 0 | NA |
| 133 | 2 | 43 | -9 | 370.4 | 106,6 | -92 | 9 | n | 0 | NA |
| 133 | 2 | 39 | -5 | 370.4 | -82,4 | -92 | 9 | n | 0 | NA |
| 133 | 2 | 32 | 1 | 370.4 | -17,4 | -92 | 9 | n | 0 | NA |
| 133 | 2 | 17 | 4 | 370.4 | -67,4 | -92 | 9 | n | 0 | NA |
| 134 | 3 | 21 | -14 | 403.5 | -14,5 | -88 | 6 | n | 0 | NA |
| 134 | 3 | 23 | -11 | 403.5 | 113,5 | -88 | 6 | n | 0 | NA |
| 134 | 3 | 18 | -7 | 403.5 | -113,5 | -88 | 6 | n | 0 | NA |
| 134 | 3 | 16 | 1 | 403.5 | 14,5 | -88 | 6 | n | 0 | NA |
| 108A | 2 | 11 | -13 | 333.5 | 177,5 | -82 | 8 | y | 7 | 15,77 |
| 108A | 2 | 10 | -10 | 333.5 | -135,5 | -82 | 8 | y | 7 | 15,77 |
| 108A | 2 | 0 | -6 | 333.5 | 111,5 | -82 | 8 | y | 7 | 15,77 |
| 108A | 2 | 1 | 2 | 333.5 | -153,5 | -82 | 8 | y | 7 | 15,77 |
| 25A | 1 | 25 | -12 | 356.4 | -100,4 | -88 | 10 | y | 10 | 15,49 |
| 25A | 1 | 34 | -9 | 356.4 | -8,4 | -88 | 10 | y | 10 | 15,49 |
| 25A | 1 | 31 | -5 | 356.4 | -94,4 | -88 | 10 | y | 10 | 15,49 |
| 25A | 1 | 39 | 1 | 356.4 | 110,6 | -88 | 10 | y | 10 | 15,49 |
| 25A | 1 | 39 | 4 | 356.4 | 92,6 | -88 | 10 | y | 10 | 15,49 |
| 29A | 1 | 8 | -11 | 296.4 | 166,6 | -80 | 8 | y | 7 | 17,09 |
| 29A | 1 | 13 | -8 | 296.4 | -6,4 | -80 | 8 | y | 7 | 17,09 |
| 29A | 1 | 10 | -4 | 296.4 | -53,4 | -80 | 8 | y | 7 | 17,09 |
| 29A | 1 | 12 | 1 | 296.4 | -46,4 | -80 | 8 | y | 7 | 17,09 |
| 29A | 1 | 12 | 4 | 296.4 | -60,4 | -80 | 8 | y | 7 | 17,09 |
| 3B | 2 | 11 | -11 | 275.5 | -23,5 | -81 | 8 | n | 0 | NA |
| 3B | 2 | 24 | -7 | 275.5 | 23,5 | -81 | 8 | n | 0 | NA |
| 46A | 1 | 4 | -11 | 307.67 | -29,67 | -87 | 9 | n | 0 | NA |
| 46A | 1 | 8 | -8 | 307.67 | -31,67 | -87 | 9 | n | 0 | NA |
| 46A | 1 | 4 | -3 | 307.67 | 61,33 | -87 | 9 | n | 0 | NA |
| 46B | 2 | 0 | -13 | 298.5 | 25,5 | -82 | 8 | y | 6 | 16,60 |
| 46B | 2 | 0 | -10 | 298.5 | -119,5 | -82 | 8 | y | 6 | 16,60 |
| 46B | 2 | 0 | -6 | 298.5 | -9,5 | -82 | 8 | y | 6 | 16,60 |
| 46B | 2 | 0 | 1 | 298.5 | 102,5 | -82 | 8 | y | 6 | 16,60 |
| 46C | 1 | 11 | -11 | 370.4 | 22,6 | -86 | 9 | y | 8 | 14,55 |
| 46C | 1 | 1 | -8 | 370.4 | 41,6 | -86 | 9 | y | 8 | 14,55 |
| 46C | 1 | 6 | -4 | 370.4 | -9,4 | -86 | 9 | y | 8 | 14,55 |
| 46C | 1 | 5 | 1 | 370.4 | 40,6 | -86 | 9 | y | 8 | 14,55 |
| 46C | 1 | 5 | 4 | 370.4 | -95,4 | -86 | 9 | y | 8 | 14,55 |
| 49B | 1 | 12 | -12 | 272.4 | -40,4 | -82 | 7 | y | 8 | 15,83 |
| 49B | 1 | 13 | -9 | 272.4 | -87,4 | -82 | 7 | y | 8 | 15,83 |
| 49B | 1 | 14 | -5 | 272.4 | 1,6 | -82 | 7 | y | 8 | 15,83 |
| 49B | 1 | 7 | 1 | 272.4 | 178,6 | -82 | 7 | y | 8 | 15,83 |
| 49B | 1 | 4 | 4 | 272.4 | -52,4 | -82 | 7 | y | 8 | 15,83 |
| 49C | 1 | 0 | -12 | 314.75 | 71,25 | -79 | 7 | y | 7 | 17,20 |
| 49C | 1 | 1 | -9 | 314.75 | -47,75 | -79 | 7 | y | 7 | 17,20 |
| 49C | 1 | 0 | -5 | 314.75 | 117,25 | -79 | 7 | y | 7 | 17,20 |
| 49C | 1 | 0 | 1 | 314.75 | -140,75 | -79 | 7 | y | 7 | 17,20 |
| 49D | 1 | 0 | -14 | 381.6 | -75,6 | -86 | 9 | y | 9 | 14,68 |
| 49D | 1 | 10 | -11 | 381.6 | -20,6 | -86 | 9 | y | 9 | 14,68 |
| 49D | 1 | 12 | -7 | 381.6 | -126,6 | -86 | 9 | y | 9 | 14,68 |
| 49D | 1 | 13 | 1 | 381.6 | -3,6 | -86 | 9 | y | 9 | 14,68 |
| 49D | 1 | 26 | 4 | 381.6 | 226,4 | -86 | 9 | y | 9 | 14,68 |
| 51A | 3 | 39 | -13 | 273.75 | 307,25 | -80 | 6 | n | 0 | NA |
| 51A | 3 | 37 | -10 | 273.75 | -102,75 | -80 | 6 | n | 0 | NA |
| 51A | 3 | 43 | -6 | 273.75 | -97,75 | -80 | 6 | n | 0 | NA |
| 51A | 3 | 27 | 1 | 273.75 | -106,75 | -80 | 6 | n | 0 | NA |
| 52B | 1 | 12 | -13 | 338.75 | -75,75 | -78 | 7 | y | 7 | 16,59 |
| 52B | 1 | 24 | -10 | 338.75 | 44,25 | -78 | 7 | y | 7 | 16,59 |
| 52B | 1 | 8 | -7 | 338.75 | 86,25 | -78 | 7 | y | 7 | 16,59 |
| 52B | 1 | 3 | 1 | 338.75 | -54,75 | -78 | 7 | y | 7 | 16,59 |
| 68E | 1 | 27 | -14 | 420.4 | 11,6 | -89 | 8 | y | 6 | 16,70 |
| 68E | 1 | 27 | -11 | 420.4 | -80,4 | -89 | 8 | y | 6 | 16,70 |
| 68E | 1 | 35 | -7 | 420.4 | 20,6 | -89 | 8 | y | 6 | 16,70 |
| 68E | 1 | 28 | 1 | 420.4 | 52,6 | -89 | 8 | y | 6 | 16,70 |
| 68E | 1 | 30 | 4 | 420.4 | -4,4 | -89 | 8 | y | 6 | 16,70 |
| 68F | 2 | 4 | -10 | 439.75 | -80,75 | -78 | 7 | y | 1 | 16,70 |
| 68F | 2 | 11 | -7 | 439.75 | 40,25 | -78 | 7 | y | 1 | 16,70 |
| 68F | 2 | 9 | -3 | 439.75 | -12,75 | -78 | 7 | y | 1 | 16,70 |
| 68F | 2 | 12 | 2 | 439.75 | 53,25 | -78 | 7 | y | 1 | 16,70 |
| 7C | 1 | 0 | -11 | 243.25 | 45,75 | -78 | 7 | y | 7 | 16,59 |
| 7C | 1 | 2 | -8 | 243.25 | 15,75 | -78 | 7 | y | 7 | 16,59 |
| 7C | 1 | 0 | -4 | 243.25 | -6,25 | -78 | 7 | y | 7 | 16,59 |
| 7C | 1 | 9 | 1 | 243.25 | -55,25 | -78 | 7 | y | 7 | 16,59 |
| 90A | 2 | 1 | -13 | 271.67 | -100,67 | -78 | 10 | y | 9 | 17,84 |
| 90A | 2 | 1 | -9 | 271.67 | 205,33 | -78 | 10 | y | 9 | 17,84 |
| 90A | 2 | 9 | -6 | 271.67 | -104,67 | -78 | 10 | y | 9 | 17,84 |
| Mar | 2 | 26 | -11 | 358.25 | 27,75 | -87 | 10 | y | 9 | 16,92 |
| Mar | 2 | 28 | -8 | 358.25 | -78,25 | -87 | 10 | y | 9 | 16,92 |
| Mar | 2 | 31 | -4 | 358.25 | 3,75 | -87 | 10 | y | 9 | 16,92 |
| Mar | 2 | 28 | 1 | 358.25 | 46,75 | -87 | 10 | y | 9 | 16,92 |
| pk44 | 2 | 18 | -10 | 187.4 | 21,6 | -75 | 7 | y | 6 | 17,47 |
| pk44 | 2 | 14 | -7 | 187.4 | -8,4 | -75 | 7 | y | 6 | 17,47 |
| pk44 | 2 | 15 | -3 | 187.4 | -23,4 | -75 | 7 | y | 6 | 17,47 |
| pk44 | 2 | 16 | 1 | 187.4 | -12,4 | -75 | 7 | y | 6 | 17,47 |
| pk44 | 2 | 24 | 4 | 187.4 | 22,6 | -75 | 7 | y | 6 | 17,47 |
| R1 | 2 | 7 | -10 | 418.8 | -73,8 | -86 | 8 | y | 8 | 15,98 |
| R1 | 2 | 20 | -7 | 418.8 | -148,8 | -86 | 8 | y | 8 | 15,98 |
| R1 | 2 | 17 | -3 | 418.8 | 82,2 | -86 | 8 | y | 8 | 15,98 |
| R1 | 2 | 19 | 1 | 418.8 | 121,2 | -86 | 8 | y | 8 | 15,98 |
| R1 | 2 | 21 | 4 | 418.8 | 19,2 | -86 | 8 | y | 8 | 15,98 |
| R10 | 2 | 13 | -12 | 375.8 | 128,2 | -87 | 8 | y | 8 | 16,38 |
| R10 | 2 | 3 | -9 | 375.8 | -33,8 | -87 | 8 | y | 8 | 16,38 |
| R10 | 2 | 0 | -5 | 375.8 | -92,8 | -87 | 8 | y | 8 | 16,38 |
| R10 | 2 | 2 | 2 | 375.8 | 118,2 | -87 | 8 | y | 8 | 16,38 |
| R10 | 2 | 1 | 4 | 375.8 | -119,8 | -87 | 8 | y | 8 | 16,38 |
| R11 | 1 | 21 | -15 | 385.6 | -49,6 | -90 | 8 | y | 5 | NA |
| R11 | 1 | 26 | -12 | 385.6 | -36,6 | -90 | 8 | y | 5 | NA |
| R11 | 1 | 29 | -8 | 385.6 | -17,6 | -90 | 8 | y | 5 | NA |
| R11 | 1 | 28 | -2 | 385.6 | 54,4 | -90 | 8 | y | 5 | NA |
| R11 | 1 | 26 | 1 | 385.6 | 49,4 | -90 | 8 | y | 5 | NA |
| R12 | 1 | 16 | -10 | 355.8 | 49,2 | -84 | 10 | n | 0 | NA |
| R12 | 1 | 31 | -7 | 355.8 | -101,8 | -84 | 10 | n | 0 | NA |
| R12 | 1 | 32 | -3 | 355.8 | 84,2 | -84 | 10 | n | 0 | NA |
| R12 | 1 | 34 | 1 | 355.8 | 72,2 | -84 | 10 | n | 0 | NA |
| R12 | 1 | 25 | 4 | 355.8 | -103,8 | -84 | 10 | n | 0 | NA |
| R13 | 2 | 1 | -12 | 366.8 | -10,8 | -89 | 12 | y | 11 | 16,01 |
| R13 | 2 | 7 | -9 | 366.8 | -172,8 | -89 | 12 | y | 11 | 16,01 |
| R13 | 2 | 18 | -5 | 366.8 | -96,8 | -89 | 12 | y | 11 | 16,01 |
| R13 | 2 | 19 | 1 | 366.8 | 180,2 | -89 | 12 | y | 11 | 16,01 |
| R13 | 2 | 26 | 4 | 366.8 | 100,2 | -89 | 12 | y | 11 | 16,01 |
| R14 | 1 | 0 | -13 | 378.75 | -20,75 | -83 | 9 | y | 9 | 17,66 |
| R14 | 1 | 4 | -10 | 378.75 | 33,25 | -83 | 9 | y | 9 | 17,66 |
| R14 | 1 | 2 | -6 | 378.75 | 147,25 | -83 | 9 | y | 9 | 17,66 |
| R14 | 1 | 5 | 1 | 378.75 | -159,75 | -83 | 9 | y | 9 | 17,66 |
| R16 | 2 | 6 | -12 | 530.6 | 185,4 | -84 | 10 | y | 10 | 17,06 |
| R16 | 2 | 19 | -9 | 530.6 | 75,4 | -84 | 10 | y | 10 | 17,06 |
| R16 | 2 | 17 | -5 | 530.6 | -49,6 | -84 | 10 | y | 10 | 17,06 |
| R16 | 2 | 13 | 1 | 530.6 | 95,4 | -84 | 10 | y | 10 | 17,06 |
| R16 | 2 | 19 | 4 | 530.6 | -306,6 | -84 | 10 | y | 10 | 17,06 |
| R2 | 1 | 5 | -10 | 330 | -8 | -90 | 10 | y | 9 | 15,82 |
| R2 | 1 | 7 | -7 | 330 | -110 | -90 | 10 | y | 9 | 15,82 |
| R2 | 1 | 14 | -3 | 330 | -68 | -90 | 10 | y | 9 | 15,82 |
| R2 | 1 | 10 | 1 | 330 | 115 | -90 | 10 | y | 9 | 15,82 |
| R2 | 1 | 26 | 4 | 330 | 71 | -90 | 10 | y | 9 | 15,82 |
| R20 | 1 | 0 | -12 | 354.75 | -58,75 | -84 | 7 | y | 7 | 16,40 |
| R20 | 1 | 0 | -9 | 354.75 | -71,75 | -84 | 7 | y | 7 | 16,40 |
| R20 | 1 | 0 | -5 | 354.75 | -70,75 | -84 | 7 | y | 7 | 16,40 |
| R20 | 1 | 0 | 1 | 354.75 | 201,25 | -84 | 7 | y | 7 | 16,40 |
| R21 | 1 | 8 | -12 | 370.6 | 55,4 | -84 | 6 | y | 6 | 17,45 |
| R21 | 1 | 15 | -9 | 370.6 | -91,6 | -84 | 6 | y | 6 | 17,45 |
| R21 | 1 | 16 | -5 | 370.6 | 88,4 | -84 | 6 | y | 6 | 17,45 |
| R21 | 1 | 28 | 2 | 370.6 | 63,4 | -84 | 6 | y | 6 | 17,45 |
| R21 | 1 | 21 | 4 | 370.6 | -115,6 | -84 | 6 | y | 6 | 17,45 |
| R22 | 1 | 16 | -11 | 358.5 | -2,5 | -92 | 7 | y | 6 | NA |
| R22 | 1 | 17 | -8 | 358.5 | -47,5 | -92 | 7 | y | 6 | NA |
| R22 | 1 | 12 | -4 | 358.5 | -39,5 | -92 | 7 | y | 6 | NA |
| R22 | 1 | 18 | 1 | 358.5 | 89,5 | -92 | 7 | y | 6 | NA |
| R23 | 1 | 28 | -13 | 374.5 | -69,5 | -87 | 8 | n | 0 | NA |
| R23 | 1 | 26 | -10 | 374.5 | -0,5 | -87 | 8 | n | 0 | NA |
| R23 | 1 | 33 | -6 | 374.5 | -70,5 | -87 | 8 | n | 0 | NA |
| R23 | 1 | 23 | 1 | 374.5 | 140,5 | -87 | 8 | n | 0 | NA |
| R24 | 1 | 0 | -11 | 465.6 | -128,6 | -85 | 9 | y | 6 | 13,33 |
| R24 | 1 | 0 | -8 | 465.6 | -49,6 | -85 | 9 | y | 6 | 13,33 |
| R24 | 1 | 0 | -4 | 465.6 | -47,6 | -85 | 9 | y | 6 | 13,33 |
| R24 | 1 | 0 | 1 | 465.6 | 85,4 | -85 | 9 | y | 6 | 13,33 |
| R24 | 1 | 1 | 4 | 465.6 | 140,4 | -85 | 9 | y | 6 | 13,33 |
| R5 | 2 | 4 | -14 | 366.6 | 72,4 | -87 | 10 | y | 4 | 17,13 |
| R5 | 2 | 15 | -11 | 366.6 | -102,6 | -87 | 10 | y | 4 | 17,13 |
| R5 | 2 | 15 | -7 | 366.6 | 38,4 | -87 | 10 | y | 4 | 17,13 |
| R5 | 2 | 1 | 1 | 366.6 | 115,4 | -87 | 10 | y | 4 | 17,13 |
| R5 | 2 | 15 | 4 | 366.6 | -123,6 | -87 | 10 | y | 4 | 17,13 |
| R6 | 2 | 0 | -12 | 324.2 | 10,8 | -90 | 11 | y | 8 | 15,14 |
| R6 | 2 | 0 | -9 | 324.2 | 7,8 | -90 | 11 | y | 8 | 15,14 |
| R6 | 2 | 6 | -5 | 324.2 | -48,2 | -90 | 11 | y | 8 | 15,14 |
| R6 | 2 | 2 | 1 | 324.2 | 102,8 | -90 | 11 | y | 8 | 15,14 |
| R6 | 2 | 12 | 4 | 324.2 | -73,2 | -90 | 11 | y | 8 | 15,14 |
| R7 | 2 | 0 | -11 | 336.6 | -78,6 | -93 | 11 | n | 0 | NA |
| R7 | 2 | 1 | -8 | 336.6 | -62,6 | -93 | 11 | n | 0 | NA |
| R7 | 2 | 1 | -4 | 336.6 | -97,6 | -93 | 11 | n | 0 | NA |
| R7 | 2 | 1 | 1 | 336.6 | 41,4 | -93 | 11 | n | 0 | NA |
| R7 | 2 | 0 | 4 | 336.6 | 197,4 | -93 | 11 | n | 0 | NA |
| R8 | 1 | 10 | -12 | 310.4 | -54,4 | -93 | 11 | y | 8 | NA |
| R8 | 1 | 25 | -9 | 310.4 | -27,4 | -93 | 11 | y | 8 | NA |
| R8 | 1 | 8 | -5 | 310.4 | -63,4 | -93 | 11 | y | 8 | NA |
| R8 | 1 | 15 | 1 | 310.4 | 171,6 | -93 | 11 | y | 8 | NA |
| R8 | 1 | 18 | 4 | 310.4 | -26,4 | -93 | 11 | y | 8 | NA |
| R9 | 1 | 3 | -11 | 415.75 | -99,75 | -94 | 11 | y | 7 | NA |
| R9 | 1 | 20 | -8 | 415.75 | 76,25 | -94 | 11 | y | 7 | NA |
| R9 | 1 | 0 | -4 | 415.75 | -60,75 | -94 | 11 | y | 7 | NA |
| R9 | 1 | 18 | 1 | 415.75 | 84,25 | -94 | 11 | y | 7 | NA |
